# The Molecular Mechanism and Therapeutic Progress in Glomus Tumor

**DOI:** 10.1002/cam4.71588

**Published:** 2026-02-15

**Authors:** Zhi Cheng Jiang, Zu Jue Cheng, Jue Xian Xiao, You Quan Huang, Zheng Ke, Jing Hui Xu, Xiang Kun Fu, Hui Huang

**Affiliations:** ^1^ Department of Neurosurgery The Second Affiliated Hospital of Nanchang University Nanchang Jiangxi China; ^2^ Fujian University of Traditional Chinese Medicine Fuzhou Fujian China

**Keywords:** gene mutations, glomus tumor, molecular mechanisms, treatment

## Abstract

**Background:**

Glomus tumor (GT) is a rare mesenchymal neoplasm presumed to originate from the neuromyoarterial glomus body. Its pathogenesis is complex and involves alterations in multiple genes and signaling pathways. In the era of precision medicine, increased molecular research has begun to elucidate the oncogenic drivers of GT, offering novel potential directions for targeted treatment strategies.

**Methods:**

This article provides a focused narrative review, synthesizing recent peer‐reviewed literature on the molecular genetics and clinical management of GT. Key findings from genetic studies, preclinical models, and clinical reports from recent years are summarized and analyzed.

**Results:**

Molecular studies have identified recurrent genetic alterations underpinning GT pathogenesis. Key discoveries include frequent inactivating mutations in the NF1 gene, leading to constitutive RAS/MAPK pathway activation, and recurrent “MIR143‐NOTCH” gene fusions disrupting Notch signaling and so on. These drivers present potential therapeutic targets. While complete surgical excision remains the standard curative treatment for localized disease, molecular insights have spurred investigation into targeted agents, including MEK inhibitors for NF1‐deficient tumors and immunotherapy. Such systemic therapies are particularly relevant for multifocal, metastatic, or surgically challenging cases.

**Conclusion:**

The integration of molecular profiling is refining the understanding of GT biology and expanding its therapeutic landscape. Moving beyond traditional surgery, the identification of targetable genetic alterations paves the way for personalized medicine approaches. Future efforts should focus on validating biomarkers in clinical trials to establish effective targeted therapies, ultimately improving outcomes for patients with complex or advanced GTs.

## 1 Introduction

Glomus tumor (GT) is a rare soft tissue tumor, accounting for < 2% of soft tissue tumors, which originates from the neuromuscular arterial device. This device is located in the subnail region of the finger, which is rich in hemangiospheres, and in the deep dermis of the palm, wrist, forearm, and foot; therefore, approximately 75% of GTs in the arm, and 65% of GTs occur in the subnail region of the distal phalanges of the fingers [1]. GT is prevalent in middle‐aged women, and its main clinical manifestations are severe pain, local pressure, and cold sensitivity disproportionate to the size of the tumor. Because of atypical clinical features, GT is often an exclusive diagnosis, and subxiphoid ultrasound and magnetic resonance imaging (MRI) technology are the main diagnostic methods, but with the increasing accuracy of ultrasound and the expensive price of magnetic resonance, more and more clinicians are choosing ultrasound as the preferred imaging method. Most GTs are benign, but a few cases exhibit histologic features of malignancy. Traditional treatments include surgery, chemotherapy, and radiotherapy. In recent years, with the rapid development of molecular medicine, an increasing number of studies have been conducted at the genetic level of GT. These currently include: (1) NF1 mutation [2], (2) NOTCH fusion [3], (3) GLMN and 1p22.1 mutations [4, 5], (4) BRAF V600E mutation [6], and (5) other gene mutations. Targeted therapies at the molecular level are also available for GT. Based on this, this article reviews recent research on GT, aiming to better understand its pathogenesis and treatment modalities.

## 2 Pathogenesis of Glomus Tumor

### 2.1 NF1 Mutation

**FIGURE 1:**
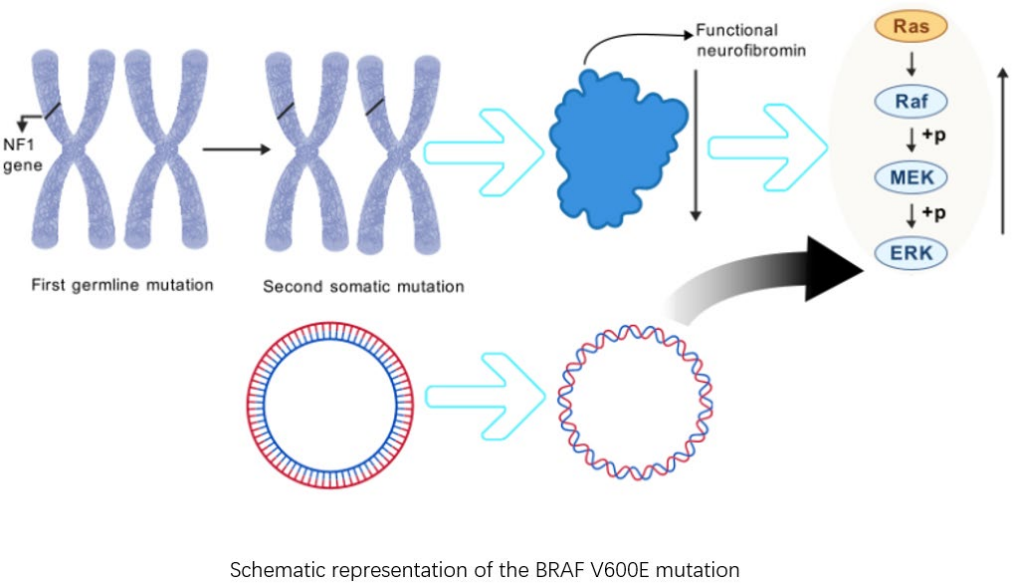
Mutations in the NF1 gene and mutations in the BRAF V600E gene together contribute to glomus tumor development through RAS‐MAPK. On the basis of germline mutations, somatic mutations in NF1 resulted in reduced expression of neurofibrillary proteins and enhanced RAS pathway activity, and mutations in BRAF V600E resulted in enhanced RAS pathway activity.

The association between NF1 mutations and GT dates back to 1938, when Robert Klaber M.D. found many bluish‐purple nodules on the trunk of a young girl with NF1, which he named “hemangioblastoid” nodules [7]. Later, in a study of eight individuals with GTs with NF1, seven were found to have multiple GTs, suggesting that the development of multiple GTs is closely related to NF1 [8, 9, 10]. In 1998, Sawada et al. speculated that extensive deletions in the NF1 gene might be related to the development of multiple GTs. Although the pathogenic gene for multiple GTs had not been identified at that time, their hypothesis could be supported by the concept of “contiguous gene syndrome” [11, 12], where the phenotype of a disease is associated with deletions of genes located near the causative gene. If the pathogenic gene for multiple GTs is located near the NF1 gene at 17q11.2, gene deletions could explain this association. Brems et al. [13] further established a causal relationship between NF1 and GTs, discovering that GTs of the fingers are part of the NF1 tumor spectrum. GTs in NF1 are secondary to bi‐allelic inactivation of the tumor suppressor gene NF1 in aSMA‐positive angioglomerular cells. In other words, NF1‐related mutations arise from germline mutations in the NF1 gene followed by somatic mutations (a “second hit”). In contrast, sporadic GT does not show NF1 mutation. Somatic gene mutations are critical events in the malignant transformation of human cells, as they can actively accelerate tumor cell growth or release the growth restrictions normally imposed on them, thereby providing a selective (proliferative) advantage at the cellular level. Stewart et al. [14] further demonstrated that mitotic recombination at the NF1 locus is the most likely cause of NF1 gene expression loss [14]. Neurofibromin (the protein product of NF1) is a negative regulator of active Ras and related Ras/MAPK signaling pathways. Neurofibromin contains a Ras‐specific GTPase‐activating protein (GAP)‐associated structural domain, which directly interacts with Ras, leading to a conformational change that greatly stimulates the intrinsic GTPase activity of Ras proteins, which significantly accelerates the conversion of the active GTP‐bound form of Ras to the inactive GDP‐bound form, and achieves a net reduction in overall pro‐mitotic signaling in cells [15]. All NF1‐associated GTs exhibit enhanced RAS‐MAPK pathway activity, so the relationship between NF1 and GT can be understood as follows: bi‐allelic mutations in NF1 lead to reduced expression of neurofibromin, weakening the protein's inhibition of the RAS pathway, thus promoting tumor formation (Figure 1).

### 2.2 NOTCH Fusion

**FIGURE 2:**
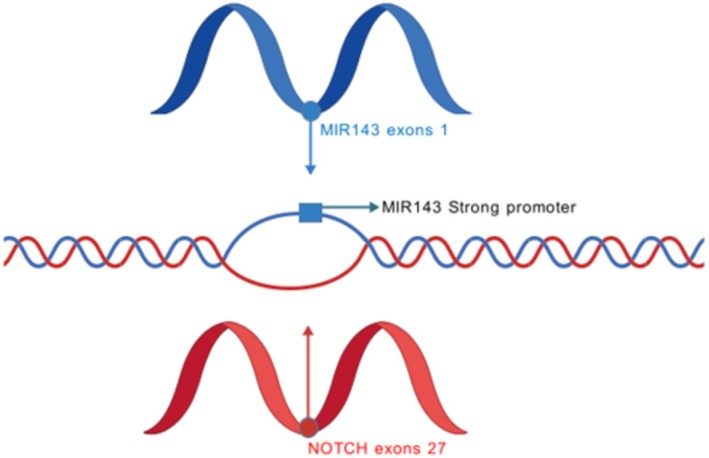
NOTCH‐MIR143 fusion causes glomus tumor; NOTCH‐MIR143 fusion, MIR143 strong promoter drives high expression of NOTCH gene.

In 2013, Mosquera et al. [3] identified the presence of MIR143‐NOTCH neofusion in more than half of GTs when attempting to classify perivascular tumors by molecular differences. NOTCH can be divided into NOTCH1–3, with NOTCH2 and NOTCH3 working together to control angiogenesis and smooth muscle differentiation [16]. The MIR‐143 genomic region contains MIR‐143/MIR‐145, which is dynamically expressed between the differentiation and proliferative phenotypes of vascular smooth muscle cells (VSMCs), and their stage‐dependent expression can cause a critical transition in VSMC phenotypic regulation. MIR‐143 and MIR‐145 have also been shown to be tightly integrated into the core transcriptional network involved in smooth muscle differentiation and proliferation, with MIR‐145 functioning as a key switch to promote smooth muscle differentiation [17]. Due to the unique plasticity of VSMCs, they can transform between proliferating or quiescent, more differentiated states. This plasticity leads to many human vascular diseases, including atherosclerosis [18, 19]. GT cells are specifically differentiated from VSMC. Due to this characteristic of VSMC, the fusion of MIR143‐NOTCH will cause its state to be abnormal. The MIR143‐NOTCH fusion pattern is the first exon on MIR 143 fusing with most of the intracellular domain (NICD) of NOTCH. NICD is the active form of the NOTCH 2 product and its high transcription rate promotes downstream gene expression, thereby promoting cell proliferation and inhibiting cell differentiation. In 2015, Nishio et al. [20] discovered a novel *t* (1; 5) (p13; q32), while NOTCH2 and MIR143 happen to be located on 1p11–13 and 5q32, respectively, a finding that strongly supports the molecular data of Mosquera et al. Narasimhan et al. also found a correlation between the molecular and clinical features of GTs. Benign GT positive for NOTCH fusion preferentially occurs in males and soft tissues of the extremities, whereas MGT with NOTCH rearrangement is more common in visceral sites. NOTCH2‐MIR143 fusion is the most common fusion companion. So far, we speculate that the pathogenesis of GT may be due to the fusion of the NOTCH gene of 1p11–13 and the MIR143 gene of 5q32, and the strong promoter of MIR143 drives the activation of the NOTCH2 pathway in GT, thereby promoting tumorigenesis (Figure 2).

### 2.3 Glomulin (GLMN) Mutation

**FIGURE 3:**
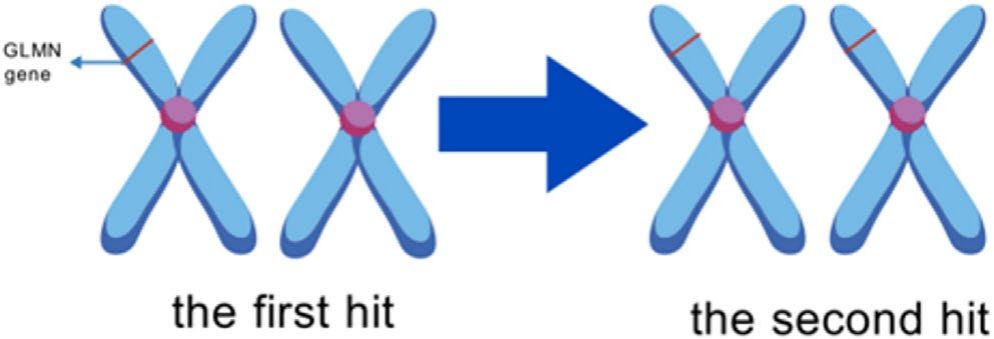
GLMN gene mutation causes glomus tumor; the second hit of the GLMN gene on chromosome 1 promotes tumorigenesis.

Bulbous vein malformation (also known as familial GT) is a genetic familial cutaneous vein injury consisting of GTs with vascular smooth muscle cell (VSMC) characteristics associated with GLMN mutations. Long before that, Brouillard et al. [21] found that some venous malformations (VMs) with inherited familial vein malformations show linkage with chromosome 9p21, resulting in ligand‐independent activation of the endothelial‐specific receptor tyrosine kinase TIE‐2. Subsequently, they did not find a TIE‐2 mutation in the glomuvenous malformation (GVM) family with VM‐like lesions in the skin associated with TIE2, but found that the GVM was genetically linked to the “VMGLOM” region of chromosome 1p21‐22. This region is approximately 4–6 Mb long and contains an estimated 200 genes, and they speculate that genetic mutations in the “VMGLOM” region may synergistically regulate angiogenesis with the TIE‐2 signaling pathway [5]. In 2001, they officially named the gene associated with GVM pathogenesis “GLMN,” which consists of 19 exons [22]. In addition, they found somatic “second hit” mutations in the affected tissues of patients with hereditary genomic deletions. Therefore, GVM is most likely caused by a complete loss of GLMN function. GLMN is similar to FAP48 (a truncated form of globin) and interacts with FKBP 12 [23]. It is known that FKBP 12 binds TGFb type I receptor (TbRI), thereby inhibiting signaling through TGFb type I receptor (TbRI) [24], and that TGFb signaling is important for both SMC maturation and the expression of SMC cytoskeletal markers. Complete loss of GLMN function would result in an increase in FKBP 12 binding to the TGFβ receptor, TGFβ signaling inhibition, altered expression of SMC proteins such as junctional proteins, and altered VSMC differentiation. The pathogenesis of GVM is similar to that of NF1‐related glomus tumors (“second hits”) [25]. The occurrence of a “double hit” of a genetic pathogenic variant could explain the emergence of GVMs, i.e., the initial change in the GLMN germline copy, followed by a second somatic hit, only with a different location of the disease‐causing gene (Figure 3).

### 2.4 BRAF V600E Mutation

The role of BRAF V600E in the pathogenesis of GTs has not been fully elucidated. In 2012 Chakrapani et al. [26] found for the first time in 28 cases of sporadic GTs the presence of three mutations in BRAF V600E, which had never been found before. It was also found that BRAF V600E‐mutated GTs signal through the Ras‐MAPK pathway, thus demonstrating its importance in the regulation of glomus growth. This is consistent with the previously described association of NF1 with glomus [13], where the loss of NF1 leads to upregulation of signaling in the mitogen‐activated protein (MAP) kinase pathway (Figure 1). They also found no significant correlation between this mutational state and the Ki‐67 index and the histological characteristics of the tumor. This is in contrast to the later study by Dashti et al. [6]. Dashti et al.'s study expands on previous work with a more detailed analysis of BRAF V600E and demonstrates that BRAF V600E‐positive GTs are significantly associated with atypical features, including deep location and large size (size > 2 cm), invasive growth, and diagnosis of malignant lesions. The sample size of the study by Dashti et al. was much larger; the study by Chakrapani et al. did not categorize GTs according to WHO criteria, and the selected GTs were basically located in the extremities where GTs tend to be benign, making the former study much more convincing. BRAF V600E mutations are most common in malignant melanoma [27] (66% of cases) and can also be found in papillary thyroid carcinoma, non‐small cell lung cancer, Langerhans cell histiocytosis, and biliary tract cancer [28, 29, 30, 31]. Although there is no genetic evidence for the pathogenic effect of BRAF V600E mutations on GT, BRAF V600E mutations are often associated with specific sites and malignant phenotypes of tumors. We conclude that three cases occurring at specific sites (1 orbit and 2 brachial plexus) all exhibited as GT‐Uncertainty of malignant potential (Table 1). Although the pathogenicity of the BRAF V600E mutation to glomus is unknown, this mutation is associated with the malignant behavior of tumors, providing a promising therapeutic target.

**TABLE 1:** Comparison of the biological behavior of glomus tumor with BRAF V600E mutation.

	Case 1 [32]	Case 2 [33]	Case 3 [34]
Location	Orbital	Lateral and posterior cords of the brachial plexus	Median nerve
Diagnosis	GT‐UMP	MGT	GT‐UMP
Size (cm)	Long 2.9	2.2 × 1.5 × 2.2	Long 4; diameter 1.5
		2.1 × 1.9 × 3.5	Long 2; diameter 0.8
Mitosis	9/50 (40×)	Increase	10/10 (400 ×)
Nuclear isomerism	—	Moderate	Mild

*Note:* — = Not performed.

### 2.5 Other Gene Mutations

**FIGURE 4:**
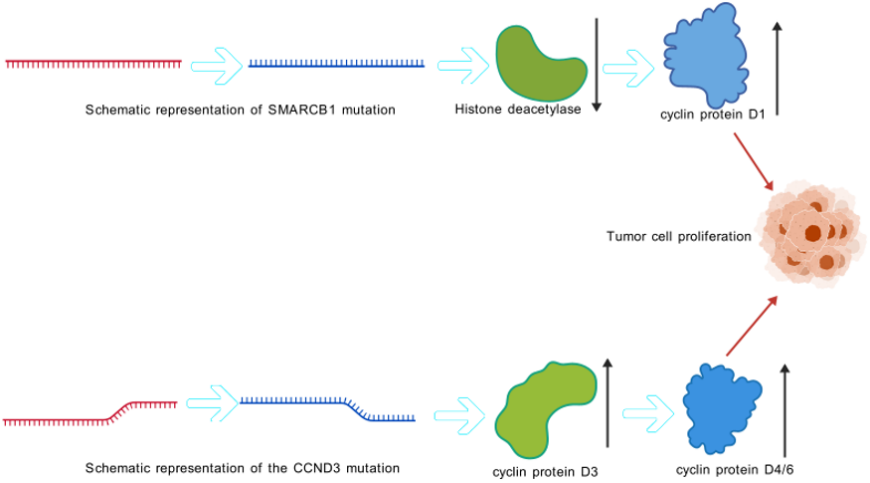
Mutations in the SMARCB1 gene decrease histone deacetylase (SMARCB1 product), leading to an increase in the cyclin protein D1; mutations in the CCND3 gene increase the cyclin protein D3, leading to an increase in the cyclin proteins D4/6; together, they promote the proliferation of glomus tumor cells.

There are also some rare genetic mutations in GT. Wojcik et al. [35] identified a new gene SMARCB1 mutation in a MGT. This gene is responsible for cell differentiation regulation, cell cycle control, and apoptosis. SMARCB1 mutations lead to depletion and reduced expression of their products, resulting in overexpression of tumor‐specific Cyclin D1 protein (Figure 4), which may be related to the development of GTs. In 2022, to evaluate the potential role of PDGFRB and NOTCH3 in the classification of pericytic tumors, Iwamura et al. [36] found that 56% of GTs have PDGFRB mutations, which are essential for the development of vascular smooth muscle cells and pericytes. Kitz et al. [37] identified a case of cutaneous MGT with a CCND3 mutation, where the CCND3 translation product affects the cell cycle by binding to cyclin‐dependent kinase 4 or 6 (CDK 4/6) (Figure 4). This is similar to the mutations that have occurred in previous MGT SMARCB1 head and neck, both of which affect cell cycle progression. In 2023, Shafi et al. [38] conducted RNA sequencing on a case of MGT from the ankle and discovered an ATG7‐RAF1 fusion, a finding previously unreported in GT. Future studies of larger cohorts are needed to determine the biological significance of these novel genes on tumors.

## 3 Treatment of Glomus Tumor

### 3.1 Surgical Treatment

Approximately 80% of GTs occur in the upper extremities, particularly in the subungual region. Surgical resection is the primary treatment method. The goals of surgery are complete tumor excision, prevention of nail deformity, and pain relief, with complete excision of the tumor being the most critical. Traditional surgical approaches, such as the dorsal nail plate approach [39], provide ample exposure and allow for maximum tumor resection. However, these methods may damage the nail bed and matrix, leading to a high incidence of postoperative nail deformity. In 2011, Roan et al. [40] proposed a new technique that involves incising both the nail and nail bed without nail avulsion, which does not require nail removal or matrix repair. This not only reduces postoperative pain but also achieves maximal tumor resection and is less time‐consuming, potentially becoming an excellent alternative method for the excision of subungual GTs. In 2020, Bae et al. [41] introduced a method of elevating the nail bed flap for the excision of subungual GTs, which can fully expose and completely resect the tumor while minimizing damage to the nail bed, thus avoiding secondary nail dystrophy and reducing postoperative pain. This method can be considered another option for the excision of subungual GTs. Vasisht et al. [42] proposed a lateral subperiosteal approach from the radial or ulnar side of the finger to reduce postoperative nail deformity. Their method creates a dorsal composite pedicle that does not invade the nail and germinative tissue, preserving normal nails. The visualization and unobstructed access for lesion removal make it suitable for any lesions occurring in that area, allowing for better and earlier postoperative activity. The main advantage of this method is the lowest incidence of postoperative nail deformity, but it is only applicable to GTs occurring in the dorsal distal phalangeal region of the finger. Although there are various surgical methods for GT, there is no consensus on which is the best, and the choice should be based on the tumor's location, size, and patient needs.

### 3.2 Chemotherapy

Given the recurrence and metastasis of MGT, chemotherapy is essential. However, due to the rarity of MGT, there is no unified standard for chemotherapy regimens, and treatment strategies often rely on those used for soft tissue sarcomas [43]. We have summarized the use of chemotherapeutic agents in GTs and their efficacy (Table 2). Based on current cases using chemotherapeutic drugs, the efficacy is variable and may be related to the type of chemotherapy drug, method of administration, and combination therapies. Compared with systemic chemotherapy, transarterial chemoembolization has the advantages of fewer side effects and greater precision, but for those MGTs that are really not surgically resectable, chemotherapy may be worth a try and may have unexpected results. In the future, prospective in vitro and in vivo studies may be needed to discover which chemotherapy regimens are more appropriate and advantageous.

**TABLE 2:** Chemotherapy in relation to glomus tumor.

Age (years)	Sex	Location	Distant metastasis	Resection	Chemical medicine	Chemotherapy efficacy
85 [44]	M	Bladder	No	Partial	Pirorubicin (TACE)	Symptom relief and tumor stabilization
44 [45]	F	Liver	No	No	Doxorubicin + ifosfamide + mesna (three cycles)	Symptomatic improvement with increased necrosis in the center of the tumor
80 [46]	F	Nasal cavity	No	No	Doxorubicin + cyclophosphamide (two cycles)	Slightly enlarged mass
55 [47]	M	Thyroid gland	No	Complete	Doxorubicin + cisplatinum	Brain and lung metastases
1 [48]	—	Neck	Double lung metastasis	No	First ifosfamide and doxorubicin (4 cycles), then vinorelbine and cyclophosphamide	Final tumor progression

*Note:* — = Not performed.

Abbreviations: F, Female; M, Male; TACE, Transcatheter Arterial Chemoembolization.

### 3.3 Radiotherapy

Although surgical resection is the main treatment for GTs, it poses a significant challenge for MGT that cannot be completely surgically eradicated. Radiation therapy is also one of our methods. Three cases [48, 49, 50] have been previously reported in which radiation therapy was applied. One case of tracheal GT that received surgical resection and adjuvant radiotherapy (total dose 30 Gy) experienced multiple recurrences and systemic metastasis; another case of the temporal region and one case of plantar GT also progressed after radiotherapy, leading to the patient's death. These cases suggest that radiotherapy not only failed to locally kill tumor cells but may have stimulated tumor cell growth. In 2023, Nam et al. [46] reported a case that showed a complete response after high‐dose radiotherapy, which was ineffective after chemotherapy. Radiotherapy on the primary tumor mass (total dose 50 Gy, divided into 20 fractions) over 4 weeks caused the tumor to almost completely regress, but unfortunately, the patient died of liver and lung metastasis 7 months later. Subsequently, Ai et al. [44] found that in a case of MGT of the bladder, the tumor significantly reduced in size after receiving 60 Gy of radiotherapy and showed no progression for 4 months. However, the tumor then invaded the anterior rectal wall, causing hematochezia. After adding chemotherapy drugs, local radioactive iodine‐125 particle implantation, and targeted therapy, the patient's symptoms were quickly relieved, and the tumor was controlled locally for a long term. We recommend an appropriate dose (e.g., 60 Gy) to eliminate tumor cells while minimizing the effects on the body. Iodine‐125 particle brachytherapy can be supplemented when the target dose is insufficient due to organ endangerment. To date, radiation therapy may provide temporary local control, and its efficacy in MGT has not been formally evaluated.

### 3.4 Emerging Targeted Therapies

#### 3.4.1 RAS‐MAPK Pathway Inhibitors

The RAS–RAF–MEK–ERK signaling pathway is a common site for oncogenic mutations, with approximately 30% of human cancers containing at least one mutation that leads to its dysregulation. The most common mutation, V600E, causes constitutive activation of the ERK pathway. Targeting the upstream genes of this pathway can reduce the expression of the signaling pathway, thereby inhibiting tumor occurrence. In 2018, Cuviello et al. [51] first reported that MGTs with BRAF mutation responded to molecular targeted therapy. They treated a case of brachial plexus MGT and considered that surgical resection might be associated with high complications, including the loss of dominant brachial plexus nerve function. Therefore, they conducted a trial of oral RAF inhibitor dabrafenib molecular targeted therapy. After combined treatment with the MEK inhibitor trametinib, the tumor intermittently shrank and remained stable after 9 months of targeted therapy. RAF kinase small molecule inhibitors were first studied in clinical trials for melanoma patients. Compared with standard chemotherapy, targeted therapy demonstrated longer progression‐free survival and overall survival [52, 53]. A phase II basket trial demonstrated modest antitumor activity against this mutation vimofenib, but not for soft tissue sarcomas [54]. For GTs that cannot be completely resected or have high complications, surgical resection can be performed after tumor shrinkage with targeted therapy. Future expansion of BRAF sequencing in malignant GTs, as well as validation of the efficacy of targeted drugs in BRAF‐mutated GTs.

#### 3.4.2 γ‐Secretase Inhibitors

In 2022, Zhang et al. [55] reported a case of long‐term clinical benefit from an orally bioavailable γ‐secretase inhibitor (LY3039478). After being diagnosed with cervical MGT, the patient's lungs metastasized following multiple surgeries, chemotherapy, radiotherapy and interventional treatments. Targeted sequencing identified the MIR143‐NOTCH1 fusion sequence in this case, and the MIR143‐NOTCH1 fusion transcript retains the NOTCH1 transmembrane domain, including the γ‐secretase cleavage site and the intracellular domain required for NOTCH1 signaling. NOTCH activation depends on a series of proteolytic cleavages, the last of which is carried out by γ‐secretase, which cleaves the NOTCH receptor within its transmembrane domain, allowing the NICD to be released from the membrane and transferred to the nucleus, forming a transcriptional activation complex. Based on this evidence, and the lack of other known systemic regimens, this patient was enrolled in a single‐case investigational new drug regimen for LY3039478, an oral γ‐secretase inhibitor. Following targeted therapy, the patient's disease was controlled and remained stable during the follow‐up period. This case expands the role of γ‐secretase inhibitors in the treatment of NOTCH‐driven tumors. In clinical trials, γ‐secretase inhibitors have shown good clinical promise for T‐cell acute lymphoblastic leukemia, lung, pancreatic, melanoma, glioma, breast, ovarian, colorectal, and sclerofibrosarcoma, but so far have only been successful in the clinical treatment of sclerofibrosarcoma and tracheal adenoid cystic carcinoma [56, 57]. This is the first report of a patient with MGT being treated with a γ‐secretase inhibitor. γ‐secretase inhibitors have good therapeutic potential for tumors in the presence of alterations in the Notch pathway, and targeted therapies are worthwhile approaches that may be worthwhile when encountering MGTs that are insensitive to both surgery and radiotherapy. Table 3 summarizes the efficacy, side effects, and indications for targeted therapy, radiotherapy, and chemotherapy.

**TABLE 3:** Comparison of three treatments for glomus tumor.

	Healing effect	Side effects	Indications
Targeted therapies	+++	Bone growth restriction, neutropenia, leukopenia, lymphopenia, oral mucositis, elevated transaminases, abdominal pain, anorexia, dry skin and knee pain, self‐limited fever, acne [51, 55]	Malignant glomus tumor insensitive to radiotherapy chemotherapy and not amenable to total surgical resection
Chemotherapy	+	—	Preoperative neoadjuvant, residual, systemic metastases, and surgically incurable malignant glomus tumor
Radiotherapy	++	Pathologic fracture [49]	Isolated, residual, recurrent, surgically incurable malignant glomus tumor

*Note:* “+” = validity, the more “+”, the more it is recommended; — = Not performed.

### 3.5 Immunotherapy

Soft tissue sarcomas are treated with total resection while ensuring a sufficient number of negative margins, followed by postoperative chemotherapy. Despite multiple therapeutic options, recurrence rates and overall survival are poor. When conventional therapy is unsuccessful, the remaining therapeutic options are limited; therefore, patients with metastatic or unresectable malignancies urgently require novel and effective soft tissue sarcoma therapy. Immune checkpoint inhibitors have recently been used to attenuate the immunosuppressive state of the tumor microenvironment in solid malignancies by restoring the immune function of T‐cells and killing the tumor cells [58]. Kazuhiko et al. [59] found that the immune markers PD‐1, PD‐L1, NY‐ESO‐1, and MAGE‐A4 were associated with their invasiveness in soft tissue sarcoma. We can also explore whether the immune markers of malignant GTs are related to their malignant behavior and prognosis, which is expected to lead to the development of targeted immunosuppressive drugs.

## 4 Conclusion

In this review, we have summarized the molecular events and treatment modalities associated with GT. While significant progress has been made in understanding the molecular mechanisms of GT, there are still gaps in knowledge. For instance, it remains unclear whether there are common pathways affected by these genes that influence the development and progression of GT, which genes can predict the malignant behavior of GT, and whether there are additional genes associated with GT that have yet to be discovered. Treatment of GT also presents challenges. Although surgical resection is sufficient for benign GT, it is not a prudent first choice for metastatic MGT. Radiotherapy and chemotherapy may play a role in treatment to some extent, and emerging targeted therapies have shown promising results. Management of MGT requires a comprehensive diagnosis and treatment, and for those that are not surgically curable, radiotherapy or targeted therapy may be given first, followed by total excision while ensuring a sufficient number of negative margins. We believe that as research into the molecular mechanisms of GT continues to advance, new therapeutic targets will be identified for the potential genetic mutations involved, leading to more scientifically informed clinical treatment plans for patients with MGT.

## Author Contributions

**Zhi Cheng Jiang:** writing – original draft. **Zu Jue Cheng:** funding acquisition, resources and writing – review and editing. **Jue Xian Xiao:** resources and writing – review and editing. **You Quan Huang:** software. **Zheng Ke:** software. **Jing Hui Xu:** software. **Xiang Kun Fu:** software. **Hui Huang:** validation, funding acquisition and writing – review and editing.

## Funding

This study was supported by Program of Jiangxi Natural Science Foundation (20242BAB25470), Jiangxi Provincial Health Commission Science and Technology grant (202110051, 202130350), Regional Projects of China Scholarship Council (2023, 43), National Natural Science Foundation of China (No. 82360475), The HealthChina·BuChang ZhiYuan Public welfare projects for heart and brain health underGrant (No. HIGHER2023099).

## Ethics Statement

The authors have nothing to report.

## Conflicts of Interest

The authors declare no conflicts of interest.

## Data Availability

The authors have nothing to report.
